# Response surface methodology-based optimisation of chitin production and its antioxidant activity from *Aspergillusniger*

**DOI:** 10.1016/j.heliyon.2024.e25646

**Published:** 2024-02-08

**Authors:** Harpreet Kaur, Deepak K. Rahi

**Affiliations:** Department of Microbiology, Panjab University, Chandigarh, 160014, India

**Keywords:** Chitin, Biomass, Yield, Optimisation, Antioxidant, RSM

## Abstract

– In this study, we focused on isolating fungi capable of producing extracellular chitin, a critical component of fungal cell walls. *Aspergillus niger* was chosen as the candidate, and we aimed to optimise chitin production. Initially, one variable at a time (OVAT) method was used to enhance chitin yield under the best fermentation conditions. Subsequently, the Plackett-Burman design was employed to identify the key medium components influencing chitin production. These factors were then fine-tuned using the Central Composite Design, resulting in the optimal concentrations of dipotassium hydrogen phosphate (0.7 mg/l), calcium chloride (0.5 mg/l), thymine hydrochloride (0.5 mg/l), and pH (4), as confirmed by ANOVA. The application of response surface methodology (RSM) led to a remarkable improvement in chitin yield, increasing it from 1.14 g/l to an impressive 4.42 g/l, a substantial 3.34-fold enhancement compared to unoptimized conditions. Additionally, we explored the antioxidant activity of the produced chitin, revealing its promising properties with a scavenging activity ranging from 32% to 55% at concentrations of 1–2 mg/ml, surpassing the control. In conclusion, our study successfully optimized chitin production from *Aspergillus niger* and demonstrated the remarkable antioxidant potential of the produced chitin, highlighting its significance in various applications.

## Abbreviations

A. niger: Aspergillus niger
OVAT: One variable at a time
PBD: Plackett Burman Design
CCD: Central composite design
RSM: Response surface methodology
DPPH: (2, 2-diphenylpicrylhydrazyl)
BHT: Butylated hydroxyl toluene

## 1 Introduction

Chitin is derived from the Greek saying ‘chiton, meaning covering or a coat of mollusc, and might have been initially utilised in 1811 by Ref. [1]. Chitin, a homopolymer of β-(1, 4, and 6) linked-acetyl d-glucosamine units, is a polysaccharide found abundantly in nature and of ultimate importance. This biopolymer is synthesised by fungi and invertebrates and is produced widely after cellulose. This natural polymer has very good properties such as biocompatibility, biodegradability, and non-toxicity. It is structurally similar to cellulose, with an acetamide group in place of the second carbon hydroxyl group of cellulose [2]. Chitin is a hard, inelastic, and white polysaccharide that occurs in nature as a component of the cell wall in plants and as microfibrils in the exoskeleton of arthropods (crustaceans, insects, and spiders). It is also synthesised by various organisms in the animal and plant kingdoms, providing strength and support to their structures [3]. The principal derivative of chitin is the fully deacetylated form, known as chitosan, which contains -(l-4)-linked glucosamine units.

The names chitin and chitosan actually refer to a family of compounds with varying degrees of deacetylation (DDA) [3]. Chitin is an important polysaccharide mainly found in fungi and arthropods. Among arthropods, chitin exists in the cuticle, shells, and backbone of insects, molluscs, and squids, respectively. Chitin is associated with proteins in fungi and animals, which have to be removed to obtain pure chitin. Chitin is present in the spores and hyphae of the fungal cell wall.

It provides the framework and structural strength in cell wall morphology, immersed in the matrix with glucan molecules in microfibril form. There is 1.2 × 10^6^ tonnes of crustacean waste produced annually, which is used as the best available source of chitin. Crustacean chitin has to be separated from minerals, lipids, proteins, and pigments. The basic method of obtaining chitin from crustacean waste involves three steps: demineralization, deproteination, and depigmentation [4]. Chitosan is also a derivative of chitin produced by deacetylation of chitin; both of these are gaining attention in many industries due to their significant advantages. Chitin is a common structural component of fungal cell walls. It is located in the innermost layer of the cell wall as microfibrils linked to mannans, glucans, and proteins [5].

Human health is deteriorated by the formation of free radicals as a result of ATP production in mitochondria. These free radicals attack the major biomolecules in the cell, such as DNA, RNA, and protein, which in turn result in hypertension, cardiovascular disease, neurodegenerative disease, and cancer. These free radicals are the direct consequences of lifestyle changes and the surrounding environment. To protect the body from these free radicals, antioxidants provide a protective effect [6]. Natural products reduce the amount of free radicals produced by environmental, toxicological, and other factors by regulating enzyme activity and signal transduction pathways, thereby exerting antioxidant activity [7]. Antioxidants may benefit human health because they protect the body from deterioration caused by free radicals [8,9]. Natural antioxidants outperform synthetic antioxidants in terms of biosafety during treatment [10]. Natural polysaccharides are biomacromolecules found in plants, fungi, algae, animals, and bacteria [11]. Natural polysaccharides contribute to their potential value in treating or preventing disease caused by oxidative stress due to their nontoxicity, stability, biodegradability, biocompatibility, and excellent antioxidant activity [12].

In the present study, the optimisation of cultural conditions for obtaining the maximum yield of chitin from an indigenous fungal isolate of soil has been performed using RSM for the improved production of fungal chitin. RSM has been used to optimise the production of biomass and chitin yield from different fungi. To the best of our knowledge, this is the first report on the optimisation of parameters for enhanced chitin production by *Aspergillus niger* using response surface methodology. Also, the antioxidant activity of chitin was evaluated using butylated hydroxyl aniline as a control.

## 2 Materials and methods

*Aspergillus niger* was isolated from the soil sample that was collected in the vicinity of Panjab University, Chandigarh, India. It was tentatively identified up to genus level after studying its cultural and microscopic features after staining with lactophenol cotton blue dye. However, identification up to species level was performed using 18S rRNA sequencing.

### 2.1 Chitin production and extraction

The extraction of chitin was done as per the method given by Ref. [13] in potato dextrose broth. The medium was autoclaved for 15 min at 15 psi pressure, and after cooling; it was inoculated with pure cultures of *Aspergillus niger* and incubated at 28 ± 0.5 °C for 7 days. At the end of the incubation period, the mycelial biomass was separated by centrifuging the fermentation broth at 5000 rpm for 10 min. It was then washed and dried. The extraction of chitin was done by taking 1 g of the dried mycelium in a clean conical flask, treating it with 10 ml of a 60% aqueous KOH/NaOH solution, and incubating it at 130 °C for 2–3 h to remove proteins, lipids, and alkali-soluble polysaccharides present in the mycelium. The insoluble material (chitin) left after KOH/NaOH treatment was washed with ethanol, dried, weighed, and preserved for further studies.

### 2.2 Optimisation studies

#### 2.2.1 Optimisation using the classical one variable at a time approach

The best chitin-producing test fungal isolate was subjected to detailed optimisation studies using the one variable at a time (OVAT) method under the best selected fermentation conditions to further enhance the chitin yield. For this, the optimisation of various cultural conditions, including physical (production medium, incubation period, temperature, pH, moisture content, inoculum size, agitation speed (rpm), and biochemical (carbon sources, nitrogen sources, carbon-nitrogen ratio, growth regulators, mineral contents), was done (Table 1). Parameters were decided as per [14,15]. All the experiments were performed in triplicates and mean of standard deviation was calculated.

**Table 1:** The various factors optimized under physical and biochemical parameters.

Medium	Potato Dextrose Broth (PDB), Malt Extract Broth (MEB), Richards's medium, Asthana & Hawker's medium, Czapek Dox medium, Brain Heart Infusion medium, Yeast extract peptone dextrose adenine medium (YPDA), Malt extract glucose yeast extract peptone (MGYP), Sabround's dextrose broth (SDA),
Incubation Days	3, 5, 7, 9, 11, 13 and 15 days
Temperature	25, 28, 30, 32, 37 and 40 °C
pH	2, 3, 4, 5, 6, 7, 8, 9 and 10
Inoculum Size	1, 2, 3, 4, 5, 6% (v/v)
Agitation Speed	50, 75, 100, 120, 150 and 200 rpm (revolutions per minute)
Carbon sources (2% w/v)	Dextrose, maltose, lactose, glucose, fructose, sucrose, galactose and mannitol
Concentration of Carbon source selected	1%, 1.5%, 2%, 2.5%, 3%, 3.5%, 4%, 4.5% and 5% (w/v)
Nitrogen sources (2% w/v)	Ammonium chloride, ammonium sulphate, urea, tryptone, peptone, yeast extract, beef extract, sodium nitrate, sodium nitrite, ammonium nitrate, potassium nitrate, cystein, casein, soyabean meal, sodium nitrate and gelatin
Concentration of Nitrogen source selected	1%, 1.5%, 2%, 2.5%, 3%, 3.5%, 4%, 4.5% and 5% (w/v)
Mineral sources (0.5 mg/l)	Dipotassium hydrogen phosphate, magnesium sulphate, manganese sulphate, ammonium chloride, sodium chloride, calcium chloride, zinc sulphate, ferrous sulphate and potassium di-hydrogen phosphate
Concentration of mineral source selected	0.1, 0.2, 0.3, 0.4, 0.5, 0.6, 0.7, 0.8, 0.9 and 1 (mg/l)
Vitamins and growth regulators (0.3 mg/l)	Indole-3-acetic acid, indole-3-butyric acid, giberallic acid, pyridoxine hydrochloride, thiamine hydrochloride, riboflavin, cycloheximide and l- glutamic acid
Concentration of vitamin and growth regulators selected	0.1, 0.2, 0.3, 0.4, 0.5, 0.6, 0.7, 0.8, 0.9 and 1 (mg/l)

#### 2.2.2 Plackett- Burman design for enhanced chitin yield

On the basis of the results of one variable at a time (OVAT) studies, the variables with the optimum concentration were selected and further processed to obtain the maximum biomass production. A set of 12 parameters was designed using Plackett-Burman and Design Expert 13 software (Stat-Ease Corporation, USA). A total of 11 variables were selected (Table 2). These 11 factors and their concentrations were selected on the basis of OVAT results. The parameters evaluated were as follows: incubation days, pH, sucrose, peptone, yeast extract, l-glutamic acid, thiamine hydrochloride, indole acetic acid, dipotassium hydrogen phosphate, calcium chloride, and ammonium chloride. All the trials were carried out in triplicate, and the chitin yield was calculated as the mean of these three values. The factors were evaluated at two levels (lower and higher). A set of 12 experiments was generated to screen the significant variables and measure their responses. The significance of the model and influence of parameters were checked by ANOVA. The factors showing the highest positive effects were selected and further optimized by response surface methodology.

**Table 2:** Plackett – Burman for screening variables at different level of ranges for Chitin production.

S. No.	Parameters	Units	Lower range	Upper range
1.	Incubation days	–	6	8
2.	pH	–	3	5
3.	Sucrose	g/l	28	32
4.	Peptone	g/l	18	22
5.	Yeast extract	g/l	18	22
6.	l-glutamic acid	mg/l	0.2	0.4
7.	Thiamine hydrochloride	mg/l	0.4	0.6
8.	Indole acetic acid	mg/l	0.2	0.4
9.	Dipotassium hydrogen phosphate	mg/l	0.6	0.8
10.	Calcium chloride	mg/l	0.2	0.4
11.	Ammonium chloride	mg/l	0.2	0.4

The effect of each parameter on response was obtained as per following equation no. 1:

(1) $$\frac{\text{Ei}=\Sigma P_{i+}\;-\;\Sigma P_{i-}}{N}$$

Where Ei is the effect of the parameter I studied. Pi_+_ and Pi- are the responses of the trials at which the parameter was studied at its high and low levels, respectively, and N is the total number of trials. Factors were analysed using the Pareto chart model. Here, factors were opened from biggest to smallest effect, i.e., from left to right, until all other factors moved above or below the Bonferroni and/or t-value limits. Factors having maximum positive effects were finally selected for optimisation using the Central Composite Design (CCD) of the Response Surface Methodology (RSM).

#### 2.2.3 Optimisation using response surface methodology

A central composite design was set up to determine the optimum values of the chosen parameters for biomass production and express their interactive effect. Four factors selected for CCD that had a positive effect in Plackett-Burman are dipotassium hydrogen phosphate, thiamine hydrochloride, calcium chloride, and pH. Factors were analysed at five different levels: low (−2, −1), medium (0), and high (+1, +2). After each experiment, responses were measured as described earlier and analysed using Design Expert version 13. Regression analysis was applied to the data obtained. Biomass production was analysed using the second-order polynomial equation no. 2. This resulted in an empirical model that linked the response calculated with the independent variables used in the experiment.

(2) Y=β0+ΣβiXi+ΣβiiXi2+ΣβijXiXj

where Y represents response variable,

β 0 is the interception coefficient,

βi, coefficient of the linear effect,

βii, the coefficient of quadratic effect and

βij, the coefficient of interaction effect.

Statistical software Design Expert 13.0.5.0 was used for ANOVA and regression analysis. Using Design Expert 13 software, contour plots and 3D plots were plotted.

### 2.3 2,2-diphenyl-1-picrylhydrazyl (DPPH) radical scavenging activity

The DPPH radical scavenging activity of the test chitin was evaluated as per [16] with a slight modification. Briefly, reaction mixtures containing 1 mL of a chitin sample (1.0 mg/mL) in 0.5% acetic acid solution, 1 mL of ethanol, and 1 mL of 0.1 mM DPPH ethanol solution will be mixed and raised to a final volume of 4 mL by 0.5% acetic acid solution in test tubes. The mixtures were mixed thoroughly and kept at 25 °C for 30 min in the dark. The absorbance of the mixtures was measured at 517 nm against a blank without DPPH using a UV–Visible spectrophotometer. DPPH radical scavenging activity was calculated using the following formula:

DPPHradicalscavengingactivity(%)=(Acontrol−Asample)/(Acontrol)×100

Where A control and A sample are absorbances of a control mixture without antioxidants and a mixture containing antioxidants, respectively.

The control taken was Butylated Hydroxy aniline.

## 3 Results and discussion

The enhanced biomass production and chitin yield from *Aspergillus niger* were achieved by optimising physical and biochemical parameters using the one variable at a time methodology. Further optimisation was done using Plackett-Burman and RSM. Three replicates were taken for each experiment.

### 3.1 Optimisation of fermentation conditions for enhancing chitin yields using OVAT

Using this approach, the various parameters affecting the chitin yield were studied in detail and analysed using single factor optimisation by keeping other parameters constant. In order to optimise the culture conditions of *A. niger*, various physical (medium, incubation days, pH, temperature, inoculum size, agitation speed) and biochemical (carbon source, nitrogen source, carbon:nitrogen ratio, vitamins, growth regulators, and mineral sources) parameters were analysed, and the results are presented in Table 3. All the parameters had positive effects. Some had minor effects, and some had major effects. Physical parameters had a minor effect, while biochemical parameters such as mineral source and growth factors had a major effect on fungal growth and chitin yield. No growth was observed with sodium nitrate or sodium nitrite. Studies revealed that organic nitrogen sources are better utilised by the fungus in comparison to inorganic sources. Many studies have reported similar results [14,17]. All the experiments were performed in triplicate, and the mean standard deviation was calculated.

**Table 3:** Optimized parameters of OVAT studies with chitin yield.

Paramters	Factors Optimized	Best Optimized Parameter	Chitin Yield (g/l)
	Medium	MEB (Malt Extract Broth)	1.15 ± 0.07
	Incubation Days	7	1.358 ± 0.14
Physical Parameters	Temperature	28 °C	1.36 ± 0.03
	pH	4	1.47 ± 0.07
	Inoculum Size	4%	1.57 ± 0.14
	Agitation Speed	120 rpm	1.6 ± 0.035
	Carbon sources (2% w/v)	Sucrose (20 g/l)	1.68 ± 0.14
	Concentration of Carbon source selected	3% (30 g/l)	1.75 ± 0.14
	Nitrogen sources (2% w/v)	Peptone(20 g/l)	1.83 ± 0.05
		Yeast Extract (20 g/l)	1.81 ± 0.14
	Concentration of Nitrogen source selected	2% (20 g/l)	1.924 ± 0.07
Biochemical Parameters	Mineral sources (0.5 mg/l)	Thiamine HCl	2.26 ± 0.03
		Glutamic acid	2.128 ± 0.07
		Indole Acetic acid	2.179 ± 0.05
	Concentration of mineral source selected	Thiamine HCl (0.5 mg/l)	2.6 ± 0.07
		Glutamic acid (0.3 mg/l)	
		Indole Acetic acid (0.3 mg/l)	
	Vitamins and growth regulators (0.3 mg/l)	K_2_HPO_4_	2.758 ± 0.07
		NH_4_Cl	2.82 ± 0.03
		CaCl_2_	2.75 ± 0.03
	Concentration of vitamin and growth regulators selected	K_2_HPO_4_ (0.7 mg/l)	3.05 ± 0.14
		NH_4_Cl (0.7 mg/l)	
		CaCl_2_ (0.3 mg/l)	

### 3.2 Selection of significant variables by Plackett-Burman design (PBD)

PBD was used to screen the significant variables for biomass production by *Aspergillus niger* under submerged fermentation. Based on the results of OVAT, eleven factors were selected, including Incubation days, pH, sucrose, peptone, yeast extract, K_2_HPO_4_, CaCl_2_, NH_4_Cl, l-glutamic acid, thymine hydrochloride, and indole acetic acid. The detailed experimental design of 12 runs for screening the significant variables along with the response is shown in Table 4. In the 12 runs, chitin yield ranged from 1.32 to 3.52 g/l.

**Table 4:** Plackett–Burman experimental design for screening important variables for the chitin production of *A. niger*.

Std	Run	Factor 1	Factor 2	Factor 3	Factor 4	Factor 5	Factor 6	Factor 7	Factor 8	Factor 9	Factor 10	Factor 11	Response 1
		A:Incubation days	B:pH	C:Sucrose	D:peptone	E:yeast extract	F:K2HPO4	G:CaCl2	H:NH4Cl	J:l-glutamic acid	K:Thymine hydrochloride	L:Indole acetic acid	Chitin Yield
				g/l	g/l	g/l	mg/l	mg/l	mg/l	mg/l	mg/l	mg/l	g/l
4	1	6	5	28	22	22	0.6	0.4	0.4	0.4	0.4	0.2	1.89
8	2	8	5	28	18	18	0.8	0.2	0.4	0.4	0.4	0.4	3.27
9	3	8	5	32	18	18	0.6	0.4	0.2	0.4	0.6	0.2	2.98
6	4	6	3	28	22	18	0.8	0.4	0.2	0.4	0.6	0.4	3.46
3	5	**8**	**3**	**32**	**22**	**18**	**0.8**	**0.4**	**0.4**	**0.2**	**0.4**	**0.2**	**3.52**
1	6	8	5	28	22	22	0.8	0.2	0.2	0.2	0.6	0.2	2.89
2	7	6	5	32	18	22	0.8	0.4	0.2	0.2	0.4	0.4	3.12
7	8	8	3	28	18	22	0.6	0.4	0.4	0.2	0.6	0.4	2.34
5	9	6	3	32	18	22	0.8	0.2	0.4	0.4	0.6	0.2	2.67
10	10	6	5	32	22	18	0.6	0.2	0.4	0.2	0.6	0.4	1.79
11	11	8	3	32	22	22	0.6	0.2	0.2	0.4	0.4	0.4	1.42
12	12	6	3	28	18	18	0.6	0.2	0.2	0.2	0.4	0.2	1.32

**Fig. 1:**
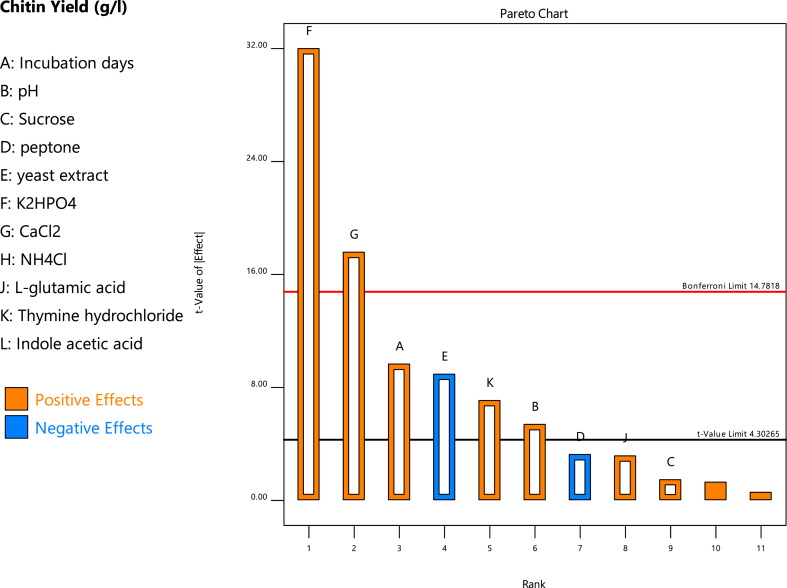
Pareto chart showing positive effect (orange colour) and negative effect (blue colour) of the different parameters used in the Placket-Burman design for Chitin production by *Aspergillus niger* under submerged fermentation.

The PBD was chosen to screen the important fermentation process components with respect to their main effects and not their interaction effects. Among the chosen variables, eight parameters showed positive effects, and only three showed negative effects. Factors showing a t-value of effect’ above the Bonferroni limit were highly significant; factors in between the Bonferroni limit and the t-value limit were moderately significant; and factors less than the t-value limit had the least effect on the response, i.e., the chitin yield (Fig. 1). The purpose of using the pareto chart was to identify statistically significant effects. Out of eleven factors tested, nine (K_2_HPO_4_ dipoatssium hydrogen phosphate, CaCl_2,_ incubation days, pH, thymine hydrochloride, l-glutamic acid, indole acetic acid, NH_4_Cl, sucrose) showed positive influences on enhancing chitin yield. Whereas, the other two factors (peptone and yeast extract) showed a negative influence on enhancing chitin yield, i.e., they did not support a much enhanced response when present beyond a certain level.

The maximum response was observed at higher levels of the parameter when the value of E*i* was positive. This indicated that the parameter was having a positive influence on the response (Orange colour bar). The maximum response was observed at lower levels when the value of E*i* was negative. This indicated that the parameter was having a negative influence on the response (Blue colour bar).

The significance of the model and the influence of parameters were checked by ANOVA. The parameters that were having a positive effect on chitin yield in the pareto chart were selected, and their p-value was calculated (Table 5). Factors with a p value less than 0.05 were considered significant and further selected for the central composite design. The factors selected were K_2_HPO_4,_ CaCl_2_, thymine hydrochloride, and pH.

**Table 5:** Variance of analysis (ANOVA) for Plackett- Burman design.

Source	Sum of Squares	df	Mean Square	F-value	p-value	
**Model**	6.77	9	0.7517	178.63	0.0056	significant
A-Incubation days	0.3924	1	0.3924	93.25	0.0106	
**B-pH**	**0.1220**	**1**	**0.1220**	**28.99**	**0.0328**	
C-Sucrose	0.0091	1	0.0091	2.16	0.2797	
d-peptone	0.0444	1	0.0444	10.55	0.0831	
E-yeast extract	0.3367	1	0.3367	80.00	0.0123	
**F–K2HPO4**	**4.31**	**1**	**4.31**	**1023.69**	**0.0010**	
**G-CaCl2**	**1.30**	**1**	**1.30**	**308.96**	**0.0032**	
J-l-glutamic acid	0.0420	1	0.0420	9.98	0.0873	
**K-Thymine hydrochloride**	**0.2107**	**1**	**0.2107**	**50.06**	**0.0194**	
**Residual**	0.0084	2	0.0042			
**Cor Total**	6.77	11				

**P-values** less than 0.0500 indicate model terms are significant. In this case A, B, E, F, G, K are significant model terms. Values greater than 0.1000 indicate the model terms are not significant.

### 3.3 Optimisation of combined effects of parameters on the chitin yield

The effects of the four independent variables (in coded form) selected from PBD design on chitin yield are shown in Table 6 along with the predicted values of effect.

**Table 6:** Central composite design showing obtained and predicted values.

Run	Factor 1	Factor 2	Factor 3	Factor 4	Response	Chitin Yield
	A:Dipotassium hydrogen phosphate	B:Calcium chloride	C:Thymine hydrochloride	D:pH	Actual Value	Predicted Value
	mg/l	mg/l	mg/l	–	g/l	g/l
1	0.7	0.1	0.5	4	3.61	3.16
2	0.6	0.4	0.4	6	3.45	3.44
3	0.7	0.3	0.5	4	3.67	4.02
4	0.7	0.3	0.5	0	3.09	2.88
5	0.6	0.4	0.4	2	2.98	3.14
6	0.7	0.3	0.5	4	4.2	4.02
7	0.8	0.4	0.6	2	2.63	2.86
8	0.6	0.4	0.6	6	2.45	2.80
9	0.7	0.3	0.3	4	3.23	3.35
10	0.7	0.3	0.5	4	4.39	4.02
11	0.8	0.4	0.6	6	2.21	2.52
12	0.6	0.2	0.4	6	2.23	2.51
13	0.6	0.2	0.4	2	2.03	2.01
14	0.5	0.3	0.5	4	1.97	1.46
15	0.8	0.2	0.4	2	2.54	2.70
16	0.8	0.2	0.6	2	2.58	2.89
17	0.6	0.2	0.6	6	1.87	2.36
18	0.8	0.4	0.4	6	3.17	3.47
19	0.7	0.3	0.5	4	3.81	4.02
20	0.8	0.2	0.6	6	2.4	2.75
21	0.6	0.4	0.6	2	2.76	3.14
22	0.6	0.2	0.6	2	2.29	2.50
23	**0.7**	**0.5**	**0.5**	**4**	**4.42**	**4.07**
24	0.8	0.2	0.4	6	3.28	3.20
25	0.9	0.3	0.5	4	2.16	1.87
26	0.8	0.4	0.4	2	3.37	3.17
27	0.7	0.3	0.5	4	3.98	4.02
28	0.7	0.3	0.5	8	3.63	3.04
29	0.7	0.3	0.5	4	4.09	4.02
30	0.7	0.3	0.7	4	3.81	2.90

All the experiments were performed in triplicates.

The predicted response for the Chitin yield can be expressed using the following second-order polynomial equation no. 3:

(3) $$\text{Chitin}\;\text{yield}=4.02+0.1042*A+0.2258*B\;-\;0.1125*C+0.0400*D\;-\;0.1650*\text{AB}\;-0.0762*\text{AC}+0.0000*\text{AD}\;-\;0.1237*\text{BC}\;-\;0.0500*\text{BD}\;-\;0.1587*\text{CD}\;-\;0.5896*A^{2}\;-\;0.1021*B^{2}\;-\;0.2258\;*\;C^{2}\;-\;0.2658\;*\;D^{2}$$

Where A is Dipotassium hydrogen phosphate, B is Calcium chloride, C is Thymine hydrochloride and D is pH.

The analysis of variance (ANOVA) of the model for the chitin yield was found to be significant (Table 7). The model's coefficient of determination (R^2^) indicated a very high correlation between experimentally obtained and predicted response values, with a R^2^ = 0.8019. This indicated that the model is good, as for a good model, R^2^ should be closed to 1.0. A maximum response of 4.42 g/l (chitin yield) was obtained with 0.7 mg/l K_2_HPO_4_, 0.5 mg/l CaCl_2_, and 0.5 mg/l Thymine hydrochloride at pH 4. With such low levels of optimum concentrations of mineral salts and growth regulators, it proved that the model was economical. The lack of fit with a p-value of 0.0549 was found to be ‘not significant, indicating that these quadratic models effectively fit the data.

**Table 7:** ANOVA results for biomass production obtained from Central Composite Design.

Source	Sum of Squares	df	Mean Square	F-value	p-value	
**Model**	13.91	14	0.9938	4.34	0.0039	significant
A-Dipotassium hydrogen phosphate	0.2604	1	0.2604	1.14	0.3032	
B-Calcium chloride	1.22	1	1.22	5.34	0.0354	
C-Thymine hydrochloride	0.3038	1	0.3038	1.33	0.2676	
D-pH	0.0384	1	0.0384	0.1676	0.6880	
AB	0.4356	1	0.4356	1.90	0.1881	
AC	0.0930	1	0.0930	0.4061	0.5336	
AD	3.553E-15	1	3.553E-15	1.551E-14	1.0000	
BC	0.2450	1	0.2450	1.07	0.3174	
BD	0.0400	1	0.0400	0.1746	0.6820	
CD	0.4032	1	0.4032	1.76	0.2045	
A^2^	9.53	1	9.53	41.62	<0.0001	
B^2^	0.2858	1	0.2858	1.25	0.2816	
C^2^	1.40	1	1.40	6.11	0.0259	
D^2^	1.94	1	1.94	8.46	0.0108	
**Residual**	3.44	15	0.2291			
Lack of Fit	3.09	10	0.3094	4.52	0.0549	not significant
Pure Error	0.3423	5	0.0685			
**Cor Total**	17.35	29				

The **Model F-value** of 4.34 implies the model is significant.

**P-values** less than 0.0500 indicate model terms are significant. In this case B, A^2^, C^2^, D^2^ were significant model terms.

**Fig. 2:**
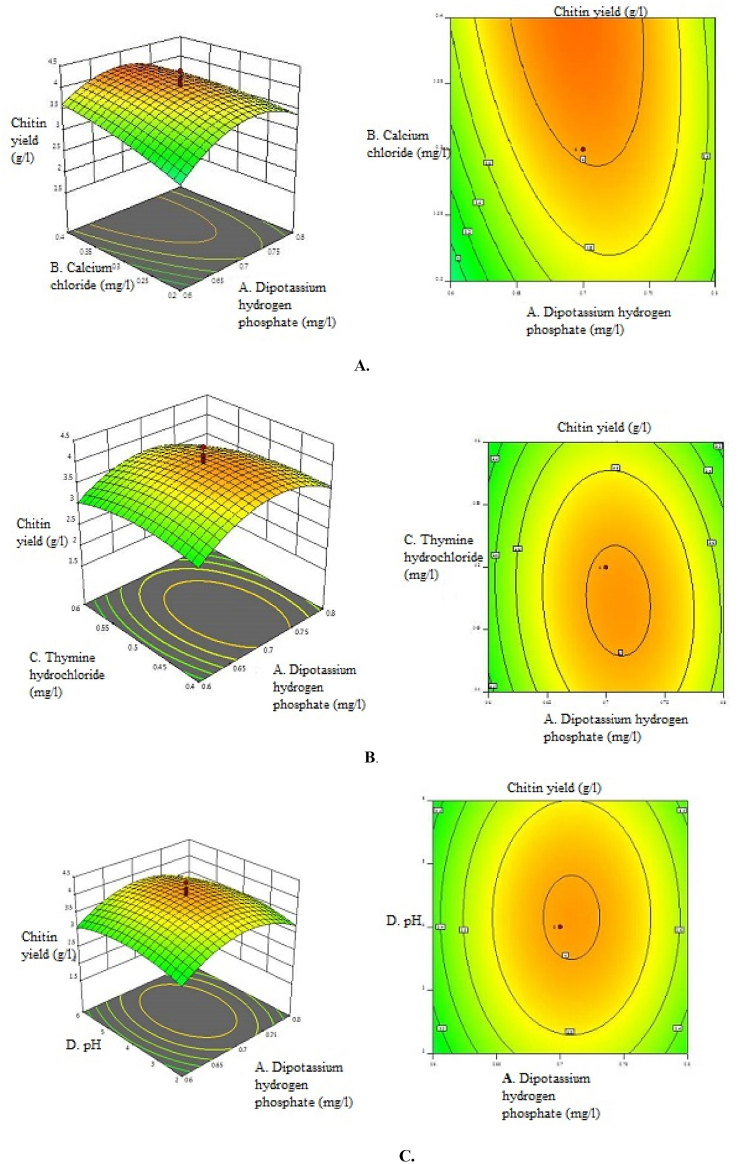

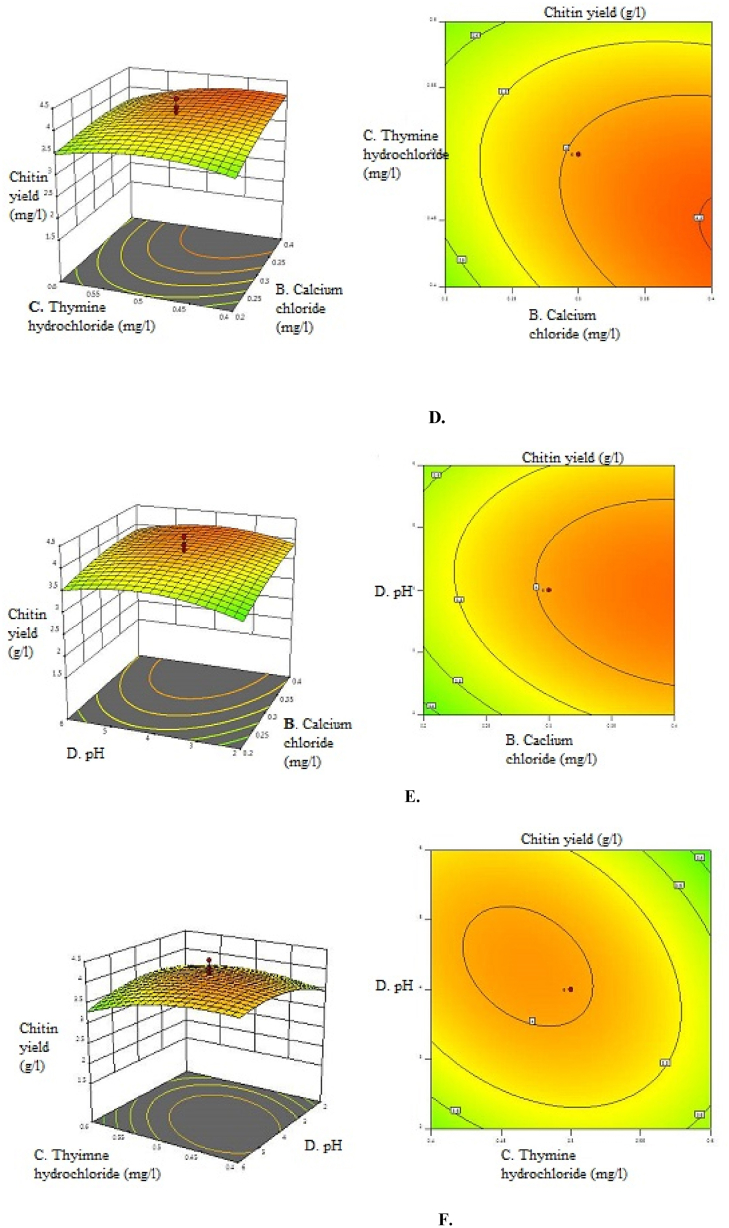
Three Dimensional response surface plots and two dimensional contour plots of A. Dipotassium hydrogen phosphate and Calcium chloride B. Dipotassium hydrogen phosphate and thymine hydrochloride C. Dipotassium hydrogen phosphate and pH D. Calcium chloride and thymine hydrochloride E. pH and calcium chloride F. Thymine hydrochloride and pH under submerged fermentation conditions.

The 3D surface plots and 2D contour plots were made to study the interaction between the four variables, and their combined effect on chitin yield from *Aspergillus niger* is presented in Fig. 2(A-F). The 3D response surface plots were generated by plotting the response (chitin yield) on the Z-axis and the two variables on the X and Y axes, whose interactions were to be studied. The response of the central point corresponded to the maximum degree of achievable chitin yield for the four factors. This data was further used to produce 3D response surface graphs and 2D contour plots by the software of Design Expert. Using these graphical plots, the values of the four parameters for maximum chitin yield were predicted. The shape of the curve indicated whether the interactions between the factors were significant or not. The maximum value for chitin yield corresponded with the central point of 3D graphs and contour plots. It was observed from 3D and the contour curves that the maximum response was located inside the design boundary, which validated the test ranges of the parameters.

The software predicted the chitin yield of 4.07 g/l using Dipotassium hydrogen phosphate, calcium chloride, thymine hydrochloride, and pH at concentrations of 0.7 mg/l, 0.5 mg/l, and 0.5 mg/l at pH 4. Therefore, the experiment was conducted using these values of variables to validate the results. A chitin yield of 4.42 g/l was achieved. The similarity between the predicted and validated response values proved the validity and applicability of the statistical model for achieving the maximum Chitin yield. A summary of the optimisation process used for enhancing the chitin yield is given in Table 8.

**Table 8:** Optimisation fold increase of chitin yield after OVAT and CCD.

Methodology used	Chitin Content (g/l)	Optimisation Fold Increase
Screening	1.14	–
OVAT	3.05	2.68
CCD	4.42	1.45

Since fungi aren't the traditional source of chitin extraction, there aren't many studies reported. There are even fewer studies available where the culture medium has been optimized to increase the chitin content of the fungi. However, there are some studies that have used a similar approach to increase the yield of chitin [18]. used the two-level factorial design to study the effect of time of cultivation and medium components like d-glucose, l-asparagine, and thymine on chitin production by *Mucor circinelloides*. The four factors showed a statistical significance of 95%. The highest chitin yield obtained was 23.9% with 60 g/l of glucose, 3 g/l of asparagine, and 0.008 mg/l of thiamine. In another study reported by Ref. [19], chitin from *C. elegans* was produced in yam bean medium with a yield of 440 mg/g after 72 h of growth. A study conducted by Ref. [20] in which three different culture media were screened for the growth of the fungus *F. solani* and its chitin content The best yield of chitin (mg per gramme of dry mycelial biomass) was obtained with Sabouraud sucrose broth for chitin (769 mg/g, or 76%).

### 3.4 2, 2-diphenyl-1-picrylhydrazyl (DPPH) radical scavenging activity of chitin

**Fig. 3:**
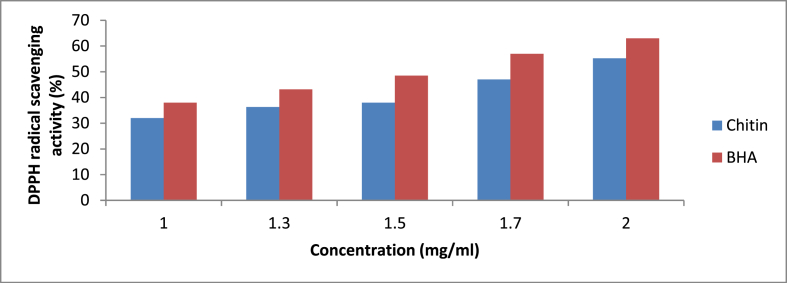
The antioxidant activity of chitin and BHA at various concentrations.

For antioxidant activity, an ethanolic extract of chitin was taken. BHA, i.e., butylated hydroxyl anisole, was used as a control. The DPPH activity of chitin and BHA at different concentrations showed slight differences (Fig. 3). The antioxidant activity of chitin is slightly lower as compared to BHA. The activity varies from 32 to 55% at concentrations ranging from 1 to 2 mg/ml for chitin, while the activity for BHA varies from 38 to 63%. A similar study using chitosan showed radical scavenging activity of 3.7–16% [21]. One study showed chitin has good antioxidant potential, obtained from the insect *Drosophila melanogaster* [22]. Another study conducted by Ref. [23] revealed the DPPH activity of chitin to be 29.6%. So our results are in correlation with previous studies conducted. Further antioxidant potential of chitin can be evaluated by total reducing power and lipid peroxidation inhibition activity.

To further advance the utilization of chitin in food applications, the purification process needs improvement, particularly considering the challenges posed by its large molecular weight and high degree of acetylation. Most previous studies have focused on chitin purification using chemical treatments such as deproteination with 1 N NaOH at temperatures ranging from 90 to 120 °C for durations ranging from 1 to 8 h, and decolorization step involving potassium permanganate [24]. However, these conventional purification methods have significant drawbacks, including high costs and environmental damage.

Some researchers have also attempted chitin purification using high performance liquid chromatography (HPLC). However, this method presents its own set of challenges. Chitin's limited solubility in many organic solvents poses a problem, and the high cost associated with HPLC equipments and consumables further restrict its practicality. The carbohydrate analysis after HPLC treatment unveiled a significant proportion of glucans (ranging from 10% to 17%). To further enhance the purity of the chitin another method has been used in a study, which included two rounds of boiling under alkaline conditions and three rounds under acidic conditions. For the alkaline boiling steps, chitin was suspended in a 5% KOH solution, while for the acid boiling steps, the resulting chitin pellets were suspended in a mixture of glacial acetic acid and 40% hydrogen peroxide in a 1:1 ratio. Remarkably, these multiple purification treatments yielded a reduction in the glucan fraction within the chitin samples. However, it is important to emphasize that the glucan content was not completely eliminated through these procedures [25].

Hence, the existing methods for purifying chitin for food applications have significant drawbacks, including high costs, environmental concerns, and technical limitations. To enhance the feasibility of chitin purification, novel approaches that address these issues should be explored.

## 4 Conclusion

In the present work, the cultural parameters for the production of chitin from *Aspergillus niger* were optimized and further validated using Response surface methodology. The most significant factors in the OVAT study were screened using Plackett-Burman, and the interaction among them was analysed using CCD. The factors consecutively affect the chitin yield from *Aspergillus niger by a* 2.68-fold increase from the initial 1.14 g/l to 3.05 g/l. To make the optimisation process more reliable and efficient, statistical experiments were conducted using response surface methodology, and it was found that the chitin yield increased from 3.05 g/l to 4.42 g/l with a fold increase of 1.45. This work further suggested that chitin yield can be further increased by altering the morphology of mycelium and by genetic engineering the organism. To the best of our knowledge, this is the first of its kind where statistical optimisation has been applied to enhance the chitin yield from *Aspergillus niger*. The antioxidant activity of chitin showed that it may be used as a functional ingredient in food formulations to improve the shelf life of food products and promote consumer health. The study indicates the potential uses of chitin as a natural antioxidant in the pharmaceutical and food industries.

## Funding

The authors did not receive support from any organisation for the submitted work.

## Data availability statement

The data associated with the present study has not been deposited into a publicly available repository. However, data will be made available on request.

## CRediT authorship contribution statement

**Harpreet Kaur:** Writing – original draft, Software, Methodology, Formal analysis, Data curation, Conceptualization. **Deepak K. Rahi:** Writing – review & editing, Supervision.

## Declaration of competing interest

The authors declare that they have no known competing financial interests or personal relationships that could have appeared to influence the work reported in this paper.
